# Three 700$ awards available from *Life Metabolism* to support conference attendance

**DOI:** 10.1093/lifemeta/load016

**Published:** 2023-04-19

**Authors:** John R Speakman

**Affiliations:** Co-EiC, Life Metabolism

The first face-to-face conference I ever attended was in the final year of my PhD. It was also the first time I had ever been abroad from the UK, and the first time I had ever flown in an airplane. It was a dizzying mix of fear and excitement. I learned that when you fly the pressure changes make you deaf for an hour. I only later learned how to cure that. I learned how to order three beers in Dutch—which is handy when you a group of three in Amsterdam, but has less utility otherwise. But most importantly, I learned that conferences are an essential part of the business of science. To that point, my interactions with other scientists consisted of my supervisor, a couple of other PhDs in his group, and the occasional visiting speaker. At the meeting, which was a small workshop held on the little island of Schiermonikoog in the Netherlands, I met about 50 other scientists working in the same area as me. It was incredible. Some of them I still know today after almost 40 years. Because this was pre-power point days, everyone came to the conference with their talks on actual slides in a carousel. There was no program. The organizer put all the carousels into a big box and then just drew them out at random to decide who the next speaker was. Every time his hand went into the box my heart rate shot up in anticipation that I would be the next. When the conference ended, I could not wait for the chance to go to the next one.

During the pandemic, attending meetings was put on hold. Strangely I gave more talks in 2021 than in any other year of my career. The reason is zoom. Having zoom and virtual attendance made it ‘virtually’ impossible to refuse to speak. After all, it is one thing to say ‘Sorry, I can't fly half way around the world and spend a weekend attending your meeting’, but another thing completely to say ‘No, I don’t have an hour 3 months from now to give a virtual presentation’. But attending a meeting on zoom is a soulless empty experience. There are no follow-up interactions with the shy but really bright student who has plucked up the courage to speak to you, and no meeting colleagues who you did not meet for 5 years. No beers on the patio discussing some confusing data. No novel collaborations that lead to papers in Science.

As we emerge from the dark days of the pandemic, we are in a different world. There are now lots of concerns about the carbon footprints of academic conferences. One recent estimate is that a 2000 attendee conference in Europe generates as much CO_2_ as 270 people generated in a whole year [1]. However, I personally think that a conference of 2000 people is way too large to be useful. Much better are smaller meetings of about 100–250 people, where there are no parallel sessions, so you can attend everything on show. Moreover, you have a reasonable chance of speaking directly to the speakers after their talks or during coffee and lunch breaks. Best of all are conferences designed to have lots of discussion and interaction time. A 4 day conference of 200 attendees only has the footprint equal to 27 people for a year. To me, that seems totally worth it. I do not mean to minimize the importance of climate issues, but this is the same carbon footprint as 200 folks going on their annual vacation, and the societal benefit of the conference seems massively greater. Plus there are often ways to mitigate the carbon costs by taking more friendly transportation options to conferences where that is feasible. Nobody should still be flying to meetings that are <500 km away. But equally if we convert everything to zoom we really lose something important, and that will slow down the progress of science. We have to find the right balance that maximizes benefit to cost ratios.

Given the enormous benefits of attending scientific meetings in person, we at *Life Metabolism* are really proud to sponsor this year Three 700 US$ prizes for students and *post docs* to help the costs of attending a meeting of their choice. These prizes have been generously sponsored by Sable Promethion China, who manufacture metabolic chambers. You can find their information via the website of Sable Systems China (sablesystems.cn). We are looking to expand this scheme in the future. So, if you are a company who wants to help us help young scientists, then please get in touch. If you are a PhD or post doc scientist who is interested in applying, then please visit the website of Life Metabolism (academic.oup.com/lifemeta) where you can find it easy to complete form to enter the competition. Good luck. We hope to make three of you happy this year, and many more in the future.
